# Use of anti–amyloid‐β monoclonal antibodies in persons with Down syndrome Alzheimer's disease

**DOI:** 10.1002/alz.71404

**Published:** 2026-04-27

**Authors:** Clíona Farrell, Fedal Saini, Mona M. Beidas, Natalie S. Ryan, Andre Strydom, Frances K. Wiseman

**Affiliations:** ^1^ UK Dementia Research Institute at University College London UCL Queen Square Institute of Neurology London UK; ^2^ Department of Forensic and Neurodevelopmental Sciences Institute of Psychiatry, Psychology & Neuroscience King's College London London UK; ^3^ Dementia Research Centre UCL Queen Square Institute of Neurology University College London London UK

**Keywords:** amyloid‐related imaging abnormalities, cerebral amyloid angiopathy, Donanemab, Lecanemab, trisomy 21

## Abstract

**INTRODUCTION:**

The recent development and licensing of anti–amyloid‐β monoclonal antibodies for the treatment of early‐stage Alzheimer's disease have significantly shifted the clinical landscape. However, current use recommendations preclude the administration of these new drugs to persons who have Down syndrome.

**METHODS:**

This narrative review considers the ethical and biological factors relating to the administration of anti–amyloid‐β monoclonal antibody therapies to persons who have Down syndrome. Literature was selected based on relevance.

**RESULTS:**

Here, we discuss the current understanding of Down syndrome Alzheimer's disease, and how this informs potential benefits and risks of treatment with anti–amyloid‐β monoclonal antibodies.

**DISCUSSION:**

The blood–brain barrier and immune system differ in persons with Down syndrome, and cerebral amyloid angiopathy is elevated compared to late‐onset Alzheimer's disease. Thus, side‐effect risks from anti–amyloid‐β monoclonal antibodies are likely to be elevated. Further research is needed to facilitate the treatment of persons with Down syndrome with these new therapies.

## Highlights

- This article provides an overview of the potential risks and benefits of the treatment of persons with Down syndrome with anti‐amyloid‐β monoclonal antibodies.
- It includes an introduction to Down syndrome Alzheimer's disease, and the clinically licensed anti‐amyloid‐β monoclonal antibodies, Lecanemab and Donanemab.
- It considers the ethics of medical treatment access, and the risks and benefits of anti‐amyloid‐β monoclonal antibodies for persons with Down syndrome, in the context of current understanding of Down syndrome Alzheimer's disease mechanisms.
- It highlights the importance of specific clinical research studies to enable the use of anti‐amyloid‐β monoclonal antibodies in persons with Down syndrome, and concludes with key considerations for this critical research.

## 1 BACKGROUND

Down syndrome (DS), caused by trisomy of chromosome 21 (Hsa21), affects around 6 million people globally, and is the most frequently occurring genetic cause of Alzheimer's disease (AD), Down syndrome Alzheimer's disease (DSAD).^1^, ^2^, ^3^ Persons with DS are identified around birth, and their greatly elevated life‐time risk of AD shortens their life expectancy.^4^ Thus, early AD treatment should be a priority for this vulnerable group of individuals. Typically, individuals with DS first accumulate amyloid‐β within neurons by their early 20s, followed by the accumulation of diffuse then neuritic amyloid‐β plaques, which precede the development of neurofibrillary tau tangles. Older individuals with DS also almost universally develop cerebral amyloid angiopathy (CAA), the accumulation of amyloid‐β in the walls of small to medium‐sized arteries and sometimes capillaries, within the brain.^5^, ^6^, ^7^, ^8^, ^9^, ^10^, ^11^ Over time, CAA displaces arteriole smooth muscle, impairing normal function and may impair blood flow and mediate microinfarcts.^12^ Individuals with DSAD may start to develop cognitive decline around age 40 and typically receive a clinical dementia diagnosis around the age of 55 years.^13^, ^14^, ^15^, ^16^, ^17^ As in the general population, age of onset varies between individuals with DSAD; however, the genetic factors driving this are not identical to those in late onset AD (LOAD).^18^, ^19^ Trisomy of chromosome 21 results in perturbations to the development and function of the brain,^20^ which may interact with the development of AD, modifying its trajectory, as suggested by preclinical research.^21^, ^22^, ^23^, ^24^, ^25^, ^26^, ^27^ This underscores the importance of considering trisomy 21 as a specific cause of AD.

Chromosome 21 encodes the amyloid precursor protein (APP), causing the overproduction of APP in persons with DS and the increased abundance of its downstream cleavage product amyloid‐β,^28^ accelerating its accumulation within the brain.^29^ Rare individuals who have partial Hsa21 trisomy, without an additional copy of *APP*, do not have accelerated plaque accumulation and appear protected from early‐onset dementia.^30^, ^31^ This supports the central role of raised *APP* gene products in the etiology of DSAD. Notably, in addition to a role of amyloid‐β, elevation of APP C‐terminal fragment‐β is linked to deleterious phenotypes in preclinical models of DSAD (Section 5).^32^, ^33^, ^34^, ^35^ Persons with autosomal dominant AD (ADAD) caused by duplication of *APP*, and persons with DSAD, both carry three copies of *APP*, but persons with DSAD also have an extra copy of all other genes on Hsa21. Comparison of these distinct genetic causes of disease highlights potential differences in biology caused by the additional copy of Hsa21 genes other than *APP*. Rates of CAA^36^ and vascular pathology^37^ are lower in persons with DSAD than in cases of ADAD caused by *APP* duplication, indicating that the extra copy of Hsa21 gene(s) carried by persons with DS may modify these aspects of AD pathology. Here, we consider the use of anti‐amyloid monoclonal antibody therapies in persons with DS, within the wider context of current understanding of DSAD mechanisms.

## 2 METHODOLOGY

This narrative review aims to consider the ethical and biological factors relating to the administration of anti–amyloid‐β monoclonal antibody therapies to persons with DS. Literature was selected by the authors based on relevance of the title and the abstract from PubMed^38^ and Alzforum^39^ using the following search terms; “Lecanemab,” “Donanemab,” “anti‐amyloid monoclonal antibod*,” “brain‐shuttle antibod*,” “amyloid‐related imaging abnormalities,” and “cerebral amyloid angiopathy” in August 2025. This evidence was synthesized to determine the following specific DS search terms conducted in PubMed in October 2025; “‘Down syndrome’ ‘health inequality’ NOT prenatal,” “‘Down syndrome’ ‘medical ethics’ NOT prenatal,” “‘Down syndrome’ ‘Alzheimer's disease’,” “‘Down syndrome’ ‘cerebral amyloid angiopathy’,” “‘Down syndrome’ cerebrovascular,” “‘Down syndrome’ Lecanemab,” “‘Down syndrome’ Donanemab,” “‘Down syndrome’ complement,” “‘Down syndrome’ ‘matrix metallo‐protease’,” “‘Down syndrome’ medin/MFGE8,” “‘Down syndrome’ immune,” “‘Down syndrome’ ‘pyroglutamate amyloid’,” “‘Down syndrome’ protofibril,” and “‘Down syndrome’ hypertension NOT pulmonary.” Additional references were also identified within the text of the relevant studies. Relevant clinical trials were identified by searching Clinicaltrials.gov^40^ using the term “Down syndrome.”

## 3 ANTI–AMYLOID‐β MONOCLONAL ANTIBODY THERAPY FOR AD

Several anti–amyloid‐β monoclonal antibodies have been trialed for the treatment of AD. Here, we focus on the two internationally licensed monoclonal antibodies, Lecanemab (Eisai, Biogen, and BioArctic) and Donanemab (Eli Lilly). Treatment of individuals with early symptomatic AD (including mild cognitive impairment and mild AD) with Lecanemab for 18 months resulted in a 27% reduction in the rate of cognitive decline compared to controls.^41^ Treatment of individuals with early‐symptomatic AD with Donanemab for 18 months resulted in a 22% reduction in the rate of cognitive decline compared with controls.^42^ A sub‐study of Donanemab in individuals with low‐moderate tau pathology showed a 37% decrease in the rate of cognitive decline, indicating that early treatment may improve outcomes.^42^ Both therapies result in a clear reduction in amyloid‐β from the brain, as measured by positron emission tomography (PET) imaging,^41^, ^42^ but the effect of treatment on long‐term cognitive outcomes is unclear,^43^ and in the short‐term are similar in effect size to symptomatic treatment with acetylcholine esterase inhibitors.^44^ Notably, individuals with DS were excluded from these industry‐sponsored trials. Both drugs have been widely licensed for the treatment of early symptomatic AD, including by the US Food and Drug Administration, the UK Medicines and Healthcare products Regulatory Agency, and the European Medicines Agency. However, despite the licenses not explicitly excluding individuals with DS, recent use recommendations for both drugs suggest they should not be used in persons with DS, which contrasts with the recommendations for ADAD, which are more nuanced.^45^, ^46^ This has resulted in these new treatments not being used in persons with DS to our knowledge, even in the United States, where Lecanemab has been available since 2023.

## 4 ETHICAL CONSIDERATIONS

Medical exclusion and health inequality are long‐standing issues for people who have intellectual disability (ID), including those with DS.^47^ This was recently illustrated during the coronavirus disease 2019 (COVID‐19) pandemic in the United Kingdom, when hospitalized individuals with ID were less likely to be intubated or admitted to intensive care.^48^ Moreover, safety uncertainty led to hesitation in COVID‐19 vaccination rollout for persons with DS, despite their greatly elevated risk of mortality caused by trisomy 21.^49^ Both paternalistic/cautious attitudes to treatment risk (protective exclusion), lack of appropriate resources, professional training, and trisomy 21–specific data contribute to these inequalities.^50^ Conversely, over‐zealous treatment in the context of a limited evidence base can cause harm, such as with the long‐term use of anti‐psychotic drugs for behavioral problems in individuals with ID.^51^ Structural issues of social discrimination and a lack of safety data further exacerbate these issues. Historical incidences of lack of informed consent, both in clinical practice and research, must also be considered. Equally, the “dignity of risk” (recognition that persons with ID have the right to make decisions that carry risk), that safety does not take precedence over autonomy, and full participation in life only occurs with the acceptance of uncertainty, should also be factored into decision making.^52^, ^53^ Recent advances in medical inclusion and communication, including person‐centered care, can circumvent these issues facilitating people with DS to be empowered to make their own decisions regarding participation in medical research and treatment, or for decisions to be made in their best interests with input from caregivers.^54^, ^55^ For example, prescribing guidelines for anti–amyloid‐β monoclonal antibodies for individuals with DSAD have been proposed.^56^ Clinical risk‐benefit assessments are based on the ethical principles of beneficence and non‐maleficence.^57^ Here, we discuss the current research evidence relating to this assessment to inform this important debate.

## 5 POTENTIAL BENEFITS ASSOCIATED WITH ANTI‐AMYLOID MONOCLONAL ANTIBODIES FOR PERSONS WHO HAVE DS

AD dementia is the leading cause of mortality for adults over the age of 35 years who have DS.^4^ Thus, effective treatments to delay or prevent AD will be of particular benefit to individuals with trisomy 21. Current data indicate that anti–amyloid‐β monoclonals only result in a modest slowing of the rate of cognitive decline in persons with AD from the general population.^41^, ^42^ The clinical value of this change has been debated,^43^, ^58^ and how this will translate to people who have DS, who have underlying ID, is unknown. Moreover, real‐world data for Lecanemab use indicates that clinical benefit may particularly occur over the age of 75 years^59^ and reanalysis of the original trial suggests the benefit may be higher in men than in women.^43^ Persons with DSAD have a much younger age of disease development but experience accelerated ageing.^60^ Moreover, the underlying disease mechanism differs from LOAD^28^ and differences in disease risk between men and women compared with LOAD have been identified.^61^, ^62^ Notably, tau pathology development may be particularly accelerated in women who have DS.^63^, ^64^ How these differences will impact monoclonal antibody efficacy in persons with DSAD is unknown.

Mechanistically, treatment with anti–amyloid‐β monoclonal antibodies results in the removal of a substantial portion of amyloid‐β from the brain.^41^, ^42^ DSAD is caused by the increased production of APP cleavage products, including amyloid‐β.^28^, ^30^, ^31^ Thus, conceptually, people with trisomy 21 should particularly benefit from the lowering of amyloid‐β. It should be noted that these treatments do not affect the level of APP C‐terminal fragments, which are also elevated in the brains of persons with DSAD.^28^, ^65^ In mouse models of DS, increased abundance of APP C‐terminal fragments are associated with the enlargement of Rab5 positive endosomes in the hippocampus, medial septal nucleus, and cingulate cortex, and an age‐dependent loss of basal forebrain cholinergic neurons.^66^ In cultured neurons isolated from a DS mouse model, elevated APP C‐terminal fragments cause raised lysosomal pH and decreased cathepsin D activity.^67^ Thus, the raised abundance of APP C‐terminal fragments that occurs in persons who have DSAD may impact neuronal health. These changes would not be modified by treatment with monoclonal antibodies targeting amyloid‐β.

A recent sub‐study of Lecanemab suggested that treatment mediates a slowing of tau pathology development.^68^ Notably, recent longitudinal studies in persons with DSAD have noted an accelerated development of tau pathology compared to AD in the general population,^69^, ^70^, ^71^ which could be mediated via a neuroinflammatory^72^ or cerebral vasculature mechanism.^73^ A large preclinical literature supports alterations in tau biology resultant from trisomy 21, independent of the effect of raised *APP* gene products.^74^ In particular, the Hsa21‐encoded kinase, DYRK1A, which has raised abundance in the brains of persons who have DS, has been shown to mediate phosphorylation of tau and indirectly modulate tau 3‐repeat/4‐repeat ratio via phosphorylation of splicing factors.^75^ Consistent with this, the abundance of phospho‐tau has been reported to be higher in samples of locus coeruleus donated by persons who had DSAD compared with samples donated by persons who had LOAD.^76^ The differences in the amyloid to tau pathology transition in persons with DSAD compared with other causes of AD could affect the efficacy and optimal treatment window for anti‐amyloid therapies.

Moreover, clinical disease progression in persons with DSAD differs from AD in the general population,^52^ and the clinical diagnostic framework differs in persons with DSAD because of pre‐existing ID.^2^ Lecanemab and Donanemab are approved for use in early‐stage symptomatic AD in the general population; determining the equivalent treatment timeframe for treatment for persons with DS requires careful judgment.

## 6 POTENTIAL RISKS ASSOCIATED WITH ANTI‐AMYLOID MONOCLONAL ANTIBODIES FOR PERSONS WHO HAVE DS

The two major risks associated with anti‐amyloid monoclonal antibodies are infusion‐related reactions and amyloid‐related imaging abnormalities (ARIA).^45^, ^46^ ARIA has two subtypes ARIA‐E (edema/effusion), and ARIA‐H (hemorrhage), and in the majority of individuals is asymptomatic. However, a proportion of individuals with ARIA develop clinical features such as headache, confusion, and gait disturbance,^41^, ^42^ and in rare cases fatal brain bleeds.^42^, ^77^, ^78^, ^79^, ^80^, ^81^ Bleeds are associated with treatment with anticoagulants^80^ or tissue plasminogen activator^78^, ^80^, ^81^ in some, but not all, cases.^42^, ^79^ Spatial and within‐patient co‐occurrence of ARIA‐E and ARIA‐H suggest common underlying mechanisms.^82^ Rates of ARIA differ between therapies, with lower rates reported for Lecanemab (ARIA‐H 14% [placebo 7.7%], ARIA‐E 12.6% [placebo 1.7%])^41^ compared with Donanemab (ARIA‐H 31.4% [placebo 13.6%], ARIA‐E 24% [placebo 2.1%]).^42^ Importantly, however, recent work has shown that a modified, more gradual titration schedule for Donanemab reduced the frequency of ARIA‐E from 23.7% to 13.7% at 24 weeks.^81^ ARIA‐E typically resolves within weeks to months after dose adjustment, suspension, or discontinuation.^83^ Infusion‐related reactions, such as fever and flu‐like symptoms, rigors, dizziness, rashes, or changes in blood pressure occurred in 26.4% of people who received Lecanemab, and 8.7% of patients who received Donanemab.^41^, ^42^ Typically, in clinical practice, an infusion reaction requires the administration of prophylactic steroids and/or antihistamines. In some centers, pre‐emptive prophylaxis has been adopted for Lecanemab administration.^84^ Several factors are proposed to modulate these risks in persons with LOAD, here we consider how these may be impacted in persons who have DS (Table 1).

**TABLE 1:** Comparison of potential risk factors relevant to the use of anti–amyloid‐β monoclonal antibodies in persons who have DSAD, compared with persons with LOAD^85^, ^86^, ^87^

Factor	DSAD	LOAD
Average age of clinical diagnosis	55 years of age^2^	>65 years of age^85^
Cause of AD	Increased production of amyloid‐β, due to a third copy of *APP* ^2^	Interaction of polygenic risk and lifestyle factors^85^
AD rate of progression	Faster than LOAD and ADAD^69^, ^70^, ^71^	Slower than DSAD
CAA prevalence	Almost universally developed, tending to be moderate to severe^5^, ^6^, ^9^, ^10^, ^11^, ^36^; prevalence increases with age^6^	Commonly developed,^86^ but less so than in DSAD
Prevalence of *APOEε4* allele	Equal to that of the general population^19^	Elevated compared to the general population^88^
Effect of *APOEε4* allele	Mild effect on disease risk^19^; no known association with CAA severity^7^, ^89^; positive association with microbleeds,^73^, ^90^ no data on ARIA risk	Strong effect on disease risk; positive association with CAA severity; positive association with microbleed and ARIA risk^45^, ^88^
Blood–brain barrier	Molecular and neuropathological changes reported^91^, ^92^, ^93^, ^94^, ^95^, ^96^, ^97^, ^98^, ^99^, ^100^, ^101^	Molecular and neuropathological changes reported^87^
Immune system	Changes to complement, central tolerance, and interferon signaling compared with the general population^102^, ^103^, ^104^, ^105^, ^106^, ^107^, ^108^, ^109^, ^110^, ^111^, ^112^, ^113^	Genetic risk linked with the innate immune system^114^
Amyloid structure	Higher levels of amyloid‐β_pE3‐X_ compared to LOAD^36^, ^115^, ^116^, ^117^, ^118^	Lower levels of amyloid‐β_pE3‐X_ compared to DSAD
Hypertension	Reduced risk compared to the general population^119^, ^120^	Elevated risk compared to the general population^85^

Abbreviations: AD, Alzheimer's disease; APOE, apolipoprotein E; ARIA, amyloid‐related imaging abnormalities; CAA, cerebral amyloid angiopathy; DSAD, Down syndrome Alzheimer's disease; LOAD, late‐onset Alzheimer's disease.

### 6.1 The risk of amyloid‐related imaging abnormalities and CAA in persons who have DS

ARIA‐risk is also associated with CAA in persons from the general population who have AD.^121^ Consistent with this, neuropathological analysis of two individuals with Lecanemab‐associated fatal ARIA showed evidence of severe CAA, macrophage infiltration, T‐cell recruitment, and significant breakdown of both plaques and vascular amyloid.^79^, ^122^ Whether the ARIA results from a direct inflammatory response to CAA or from antibody‐mediated movement of amyloid‐β from plaques to the perivascular space, or a combination of the two, is unclear.^82^, ^123^

Individuals who have DSAD have an age‐dependent elevated risk of CAA, with occurrence higher than LOAD, equivalent to ADAD (caused by *APP/PSEN1/PSEN2* mutations), and lower than in ADAD caused by *APP* duplication.^5^, ^6^, ^7^, ^9^, ^10^, ^36^ Neuroimaging studies demonstrate a high prevalence of CAA‐related markers in persons with DS, including white matter hyperintensities, enlarged perivascular spaces, and cerebral microbleeds.^124^ These first appear in the mid‐to‐late thirties, showing strong age‐related increases, and association with AD‐stage^125^ with earlier and more severe progression compared to the general population.^89^ These factors are also associated with ARIA risk.^45^, ^46^ Cerebrovascular markers also contribute to clinical progression in persons with DSAD, both independently and synergistically with the effect of AD neuropathology,^126^ highlighting the clinical importance of these changes.

Notably, individuals younger than 40 years of age who have DS, typically do not have substantial CAA, as detected by neuropathological examination.^5^, ^10^, ^11^ Moreover, in neuropathological studies of tissues donated by persons who had DSAD, age is a predictor of CAA severity.^6^ Consistent with neuroimaging studies, microbleeds detected by neuropathological methods are first observed in the occipital cortex after the age of 30, and then around a decade later in the frontal cortex in persons with DS.^9^ Importantly, this study also indicated that microbleeds are associated with the presence but not the severity of CAA. These data suggest that younger persons with DS, particularly those under 30, may have a reduced risk of side effects from anti–amyloid‐β monoclonal antibodies compared with older individuals. Current usage recommendations cite the elevated risk of CAA and associated risks for ARIA in people who have DS as the major reason not to use anti‐amyloid monoclonal antibodies.^45^, ^46^

### 6.2 The risk of ARIA and *APOE* genotype in persons who have DS

Monoclonal antibody ARIA risk is associated with the *APOEε4* genotype in persons who have LOAD.^80^, ^127^, ^128^ Moreover, in the general population *APOEε4* elevates the risk of LOAD, such that around half of all people with LOAD carry at least one copy of this risk allele.^88^ Notably, persons who have DSAD do not have an increased frequency of the *APOEε4* allele compared with the overall population, such that only around 20% of persons who have DSAD carry one or more copies of this allele.^19^ Therefore, on average at a group level, people who have DSAD may have a lower *APOEε4*‐driven risk of ARIA than persons with LOAD. Furthermore, in the general population, *APOEε4* genotype is associated with the incidence and severity of CAA.^129^, ^130^ Consistent with this, in a neuropathological study of CAA, a relationship between the *APOEε4* allele and CAA severity was observed in persons who had LOAD, however, no relationship between *APOEε4* and CAA severity was found in samples from persons who had DSAD.^7^ Similarly, the absence of an effect of the *APOEε4* allele on neuroimaging markers of CAA has been reported for persons with DS.^89^ However, recent imaging studies of persons with DS demonstrated an association of *APOEε4* and an elevated risk of microbleeds.^73^, ^90^ In persons with DS microbleed risk also increases with age and dementia status,^73^, ^90^, ^125^, ^131^ highlighting the importance of further research understanding how the *APOEε4* allele influences CAA development and ARIA‐risk in persons who have DS, and how these interact with age and dementia.

### 6.3 The risk of ARIA, vascular changes, and the blood–brain barrier in persons who have DS

In addition to elevating the risk of CAA, trisomy 21 may also modify other aspects of ARIA risk. The mechanisms that underlie ARIA are not well understood but may relate to the immune and vascular systems.^82^, ^123^ Genetic risk, clinical, and imaging similarities between ARIA and CAA‐related inflammation (CAAri) suggest common underlying mechanisms.^132^ CAAri can occur spontaneously in individuals without pre‐existing cognitive impairment or can occur rarely during the course of LOAD or ADAD.^133^ Spontaneous CAAri is relatively rare and understudied and is linked with amyloid‐β auto‐antibodies.^134^, ^135^ Treatment with steroids appears to be beneficial,^136^ although spontaneous improvement can also occur. Individuals with DS have been reported to have elevated levels of amyloid‐β auto‐antibodies,^137^ and a case report of CAAri in a woman who had prodromal DSAD (aged 59) was recently identified.^138^ Moreover, case reports of CAA‐associated hemorrhage,^139^, ^140^, ^141^, ^142^, ^143^, ^144^ and age‐dependent elevated rates of microinfarcts, intracranial hemorrhage, and cortical superficial siderosis in adults with DS^73^, ^89^, ^90^, ^125^, ^131^ have raised concerns regarding ARIA risk in persons who have DS.

Matrix metalloproteases 2/9 (MMP2/9) are expressed in blood vessels and have been suggested to have a role in CAA‐related hemorrhage.^145^ Thus, consideration of how trisomy of Hsa21 affects the biology of these enzymes is pertinent to assessing ARIA risk in individuals with DS. Increased MMP9 is observed in the occipital cortex in persons without DS who had CAA‐associated hemorrhages, compared with persons who had CAA but no evidence of hemorrhage.^146^ Elevated MMP2 is observed in regions of the brain affected by hemorrhage compared with the unaffected contralateral region of the brain in people who had extensive CAA.^147^ MMP9 abundance in the occipital cortex of persons with CAA is also found to correlate with CAA severity.^148^ In wildtype mice, and in mouse models of CAA, MMP9 treatment leads to raised rates of intracranial hemorrhage, and has been suggested to have a causal role in vascular remodeling, breakdown, and bleeding.^148^ Raised MMP9 abundance occurs throughout the cortex and in cerebrospinal fluid (CSF) in individuals who have DSAD compared to healthy ageing.^91^, ^92^ MMP2 (and associated proteins) are elevated in CSF before changes in typical AD biomarkers in persons with DS.^93^ Whether these raised MMP levels are a cause or consequence of CAA, and what the potential impacts on hemorrhage and ARIA risk for the DSAD population, is unknown.

In addition to the observed changes in MMP abundances in persons who have DS, other aspects of brain‐blood‐vessel biology, including in brain endothelial cells, are also altered by trisomy of Hsa21. These changes in the brains of people who have DS may also impact ARIA‐risk. For example, the LOAD risk gene, metalloprotease *ADAMTS1*,^114^ which is located on Hsa21, is upregulated in the brains of persons who had DSAD,^94^ particularly in endothelial cells.^65^ The impact of this on CAA and ARIA in people with DSAD has yet to be investigated. The MFGE8 cleavage product, medin, is also linked with CAA.^149^, ^150^ MFGE8 is decreased in the CSF of persons with DS,^93^ but medin levels have not been reported. The cause and consequence of alterations to MMP, ADAMTS1, and MFGE8 abundance, and the impact of this on ARIA risk in persons who have DSAD warrants further investigation.

Changes to blood–brain barrier (BBB) integrity which occur in people who have DS may also contribute to ARIA risk. Notably, proteomic data demonstrate an impairment in the BBB in adults with DS,^93^, ^95^ consistent with reports of the ingress of plasma components into the brain in persons with DSAD.^96^, ^97^ Differences in aquaporin 4 abundance and cellular distribution in the brains of persons with DS compared with individuals with LOAD,^98^ breakdown of the BBB (fibrinogen leakage), and a reduction in the number of pericytes in individuals with DSAD compared with neurotypical controls have been reported,^99^ further indicating that the BBB may be altered in persons who have DS. An early neuropathological study using lectins, comparing samples from persons with DSAD with those from the general population with AD, indicated that changes to the BBB only occur in individuals with DS over the age of 50.^100^ A small neuropathological study indicated that loss of vessel density is similar in persons with DSAD compared to that in individuals with LOAD, although significant variation between brain regions and cases was observed.^101^ These data collectively indicate that the BBB is perturbed in persons with DS, particularly in older individuals or those who have more extensive AD neuropathology. This may contribute to the risk and progression of ARIA in persons with DS.

### 6.4 The risk of ARIA, infusion‐related reactions, and the immune system in persons who have DS

Neuropathological evidence supports an association between CAA and complement components, including between membrane attack complex components and arteriole‐CAA,^151^, ^152^ and raised serum C3 in individuals who had MCI and CAA imaging markers, compared with those who did not.^153^ This suggests that a complement‐mediated CAA inflammatory response may contribute to ARIA. Complement biology is dysregulated in people who have DS; changed component abundance,^102^, ^103^, ^104^ and complement gene transcription,^105^, ^106^ and an association of complement with amyloid‐β plaques^107^ has been reported. This dysregulation of complement signaling may impact the risk and progression of ARIA in persons who have DS.

Recruitment of peripheral immune cells is linked to severe ARIA.^77^, ^79^ The peripheral immune system is altered in people who have DS, changing the response to infection, vaccination, and elevating autoimmune disease risk^108^ likely because of breached central‐tolerance^109^, ^110^ and enhanced interferon signaling.^111^, ^112^, ^113^ How these differences impinge on ARIA and infusion‐related reactions is unknown. Notably, a recent conference report linked CAA with a type 1 interferon response.^154^ Other monoclonal antibodies (including anti–tumor necrosis factor‐α, anti‐interleukin12, anti‐interleukin17, and anti‐interleukin23 biologics) have been safely administered to people who have DS, for the treatment of DS‐associated arthritis and autoimmune dermatological conditions, including hidradenitis suppurativa.^155^, ^156^, ^157^ However, rates of infusion‐related reaction have not been reported in these studies. Relatively lower efficacy of first‐line biologics targeting tumor necrosis factor‐α has been reported for the treatment of arthritis in children with DS compared with the typically developing population.^158^ If differences in the immune systems of persons who have DS will affect the safety and efficacy of Lecanemab and Donanemab is unknown.

### 6.5 The risk of ARIA and amyloid structure in persons who have DS

Differences in the efficacy and risk of ARIA between monoclonal antibodies may result from the conformation and/or subtype of amyloid‐β targeted. Lecanemab targets protofibrils^159^ and has a particular affinity for small aggregates.^160^ Although differences in the distribution of amyloid‐β aggregates and novel structures occur in brain samples from persons who had DSAD, particularly in older individuals,^161^, ^162^ a similar profile of *post mortem* neuropathology Lecanemab binding is reported for persons who had LOAD or DSAD.^163^ Moreover, in neuropathological samples from persons who had DSAD, widespread binding of a Lecanemab‐biosimilar antibody to both plaques and CAA occurs.^164^ Donanemab targets N‐terminally truncated pyroglutamate amyloid‐β (amyloid‐β_pE3‐X_).^165^ In persons with DS, amyloid‐β_pE3‐X_ is found in both plaques and CAA.^36^, ^115^ Consistent with this, Donenemab is observed to bind to a subset of plaques in neuropathological samples from persons who had DSAD.^166^ Moreover, a higher burden of amyloid‐β_pE3‐X_ occurs in neuropathological samples from persons who had DSAD compared to samples from individuals who had EOAD,^116^ or LOAD particularly in the cerebellum.^117^ A biochemical study comparing frontal cortex and precuneus samples from persons with DSAD and LOAD indicates this may be driven by raised amyloid‐β_40_, rather than elevation of pyroglutamate‐modification rate.^118^ Additionally, a high CAA burden in the cerebellum has recently been reported to occur in persons with DSAD.^8^ These data indicate that the efficacious Donanemab dose may differ in persons with DSAD, and that the risk of ARIA in the cerebellum as well as the occipital cortex should be particularly considered during Donanemab treatment.

### 6.6 The risk of ARIA and blood pressure in persons who have DS

A retrospective analysis of Donanemab‐ARIA risk has highlighted hypertension as a risk factor.^128^ Individuals who have DS have a reduced risk of hypertension,^119^ but as in the general population this risk rises with age.^120^ In persons with DS, hypertension is associated with white matter hyperintensities but not microbleeds or infarcts.^131^, ^167^ Notably, the pattern of cerebral microbleeds in persons with DS differs from that in the general population, with microbleeds in the cerebellum and occipital cortex predominating,^131^ further emphasizing the importance of monitoring these regions for ARIA in persons with DS. In persons with DS microbleed risk increases with age, dementia status, and *APOEε4* allele positivity,^73^, ^90^, ^125^, ^131^ as in the general population. Therefore, *APOE* genotype and age are paramount safety considerations for the use of anti–amyloid‐β antibodies in persons with DS.

## 7 CLINICAL TRIALS FOR PERSONS WITH DSAD

The uncertainty regarding both the benefit and risk of anti–amyloid‐β monoclonal antibody treatment for persons who have DS highlights the need for specific clinical trials to determine the safety and efficacy of these new drugs in this population. Currently, a planned trial, Amyloid Lowering for Alzheimer's in Down's with Donanemab Investigation (ALADDIN) will investigate Donanemab safety and efficacy, using brain amyloid‐PET imaging as a read‐out, in 60 adults with DS who have evidence of amyloid‐β pathology. The exclusion criteria of persons who are homozygous for *APOEε4* and those with pre‐existing immunological or inflammatory conditions are prudent, given the heightened safety risks. The study is not powered to determine clinical efficacy and the main cognitive measure is a memory test (modified Cued Recall Test [mCRT]) that has limited broader clinical applicability and may exclude people with more pronounced ID who do not achieve adequate baseline scores from inclusion, thus, limiting the application of findings from this initial study to all persons with DS. To establish benefit and whether risks are justified, a larger trial may ultimately be required.

New Brain‐Shuttle antibodies, such as Trontinemab (Roche), in which the monoclonal antibody (gantenerumab) is conjugated to a BBB shuttling receptor (transferrin receptor target), are proposed to mediate specific BBB uptake and prevent early immune activation, enhancing safety.^168^, ^169^ Early Phase II trial data reported relatively low rates of ARIA, but the full data have yet to be peer‐reviewed, and Phase III trials are ongoing.^170^ This class of antibodies may therefore have particular value for the treatment of persons with DSAD, given the changes to the immune system and higher rates of CAA that occur in persons with DS. However, as the treatment is given via intravenous infusion, it may not be suitable for all individuals with DS. Moreover, no brain‐shuttle antibody trials are currently recruiting individuals with DS, thus, specific efficacy and safety data for trisomy 21 is lacking.

In addition, other novel AD therapies may also benefit persons with DSAD. Recently, two trials of alternative amyloid‐targeting treatment for adults who have DS were open for recruitment.^171^ The ABATE trial (AC‐Immune) is an ongoing Phase Ib/II trial studying the effects of ACI‐24.060, an active immunotherapy targeting amyloid‐β oligomers and fibrils, in both individuals with DS and those with prodromal AD. The treatment is given via intra‐muscular injection. In preclinical animal studies, ACI‐24 induces a polyclonal antibody response against both oligomeric and pyroglutamate amyloid‐β species.^172^ A small early‐phase study of an earlier form of this treatment in persons who had mild AD did not report ARIA nor include frequent imaging of participants.^173^ The HERO trial (Ionis Pharmaceuticals) was an early phase trial recruiting adults with DS to evaluate the safety, tolerability, and pharmacokinetics of an antisense oligonucleotide therapy (ION269) given intrathecally to reduce amyloid‐β by silencing *APP*. However, this study recently closed to recruitment before the planned end date, reportedly because of a low recruitment rate. The low recruitment rate may have been the result of participant concerns regarding the intrathecal administration route. This highlights the importance of careful consideration of the route of delivery of novel therapies for persons who have DS.

## 8 CONCLUSIONS

The BBB and immune system differ in persons with DS, and CAA is elevated compared to individuals who have LOAD. Thus, it seems likely that the side‐effect risk from anti–amyloid‐β monoclonal antibodies will be elevated in persons who have trisomy 21. To mitigate treatment risk, treatment of individuals at an age when CAA is likely to be minimal, with careful monitoring for early signs of ARIA, particularly in the cerebellum and occipital lobes, infusion‐related reaction management planning, screening for immune dysregulation, careful dose selection, and dose escalation should all be considered. We welcome the recent commencement of clinical trials for AD‐therapies in persons with DS and the additional information these studies will provide. We highlight the importance of further DSAD‐focused clinical research to facilitate the use of novel AD treatments in persons with DS, including the need to better understand neurovascular and neuroimmune biology in persons with DS, and how these change with age and AD neuropathology development.

## CONFLICT OF INTEREST STATEMENT

The authors declare that they have no competing interests. Frances K. Wiseman has undertaken for fee consultancy for Alnylam Pharmaceuticals and TRIMTECH Therapeutics. Author disclosures are available in the Supporting Information.

## Supporting information



Supporting Information
